# Poor sleep quality and its associated factors among working adults during COVID-19 pandemic in Malaysia

**DOI:** 10.1017/gmh.2024.23

**Published:** 2024-03-13

**Authors:** Lwin Mie Aye, Wei Hao Lee

**Affiliations:** 1 Public Health and Community Medicine Department, School of Medicine, International Medical University, Kuala Lumpur, Malaysia; 2 South East Asia Community Observatory (SEACO) and Global Public Health, Jeffrey Cheah School of Medicine and Health Science, Monash University, Selangor, Malaysia; 3 School of Medicine, International Medical University, Kuala Lumpur, Malaysia; 4 School of Medicine, University of Edinburgh, Edinburgh, UK

**Keywords:** sleep quality, COVID-19 MCO, working adults, PSQI, Malaysia

## Abstract

**Background:**

In Malaysia, a nationwide movement control order (MCO), implemented to curb the COVID-19 spread, impacted on the lives of the working population which could impair sleep quality.

**Objective:**

This study aims to find the sleep quality status and its association with the socioeconomic, employment and lifestyle factors of working adults during the MCO period.

**Methods:**

A cross-sectional study was conducted among 500 eligible working adults. Data were collected using a structured questionnaire consisting of the Pittsburg Sleep Quality Index.

**Results:**

The proportion of poor sleep quality is found to be 59.4%. Analysis shows that the use of electronic devices before sleep (OR = 2.33, 95% CI = 1.02–5.35, *p*-value = 0.046), increased amount of workload (OR = 0.45, *p*-value = 0.005), decreased in amount of workload (OR = 0.48, *p*-value = 0.003) and distracted while working (OR = 0.57, *p*-value = 0.014) are the factors significantly associated with and are predictors of poor sleep quality.

**Conclusion:**

During crisis, there is a need for public health interventions for the working population to adopt a healthy lifestyle. Employers are recommended to support employees’ well-being and to provide a healthy workplace during challenging times. Policy recommendations are also made to implement flexible working arrangements, workload management, workplace mental health support and legal protections on reasonable working hours, rest breaks and time off during crises.

## Impact statement

Movement control order (MCO), implemented during the COVID-19 pandemic, results in job instability in Malaysia where many businesses and companies struggling to function and many economic sectors facing revenue losses. Factors ranging from fear of infection to monetary loss among the working population have led to a significant impact on mental health, giving rise to poor sleep quality. Studies have been done to explore the sleep quality among healthcare workers and university students, and the results were poor. This has yet to be explored among the general working population. During MCO, the majority of the working adults had to work from home and struggle between family and work. This compromises their normal routines that have impacts on their food intake and lifestyle. As a result, the sleep quality of the working population could be impaired. In this study, it was found that the proportion of poor sleep quality among the working population is found to be high. The results show that getting more sedentary, use of electronic devices before sleep, changes in the amount of workload, and being distracted while working during the MCO are the factors that are associated with poor sleep quality among the working population in Malaysia. The findings highlight that during the period of crisis, there are need for public health interventions to empower the working population to adopt a healthy lifestyle, achieve the required level of physical activities and provide sleep hygiene education. The findings provide employers with the evidence to provide measures to support employees’ well-being during challenging times.

## Introduction

The COVID-19 pandemic started in the year 2019 and has quickly become a global health emergency. The impacts of the COVID-19 pandemic and consequent lockdowns worldwide have been well observed over the months, and Malaysia, just like other countries, entered a nationwide movement control order (MCO) to curb the spread of viral infection (Aziz et al., 2020; Law, 2020). This results in job instability in Malaysia, where many businesses and companies struggling to function and many economic sectors facing revenue losses (Chopra et al., 2020; Lai et al., 2020). A survey published in December 2020 by the World Bank Group stated that a large revenue drop of 25% was observed for Malaysia during MCO (Kuriakose and Tran, 2020). Factors ranging from fear of infection to monetary loss among the working population have led to a significant impact on mental health, giving rise to stress, anxiety and distress (Lai et al., 2020; Rossell et al., 2021). Moreover, because of MCO, the majority of the working adults had to work from home, struggle between family and work and compromise their normal routines, which had impacts on their food intake and lifestyle (Chopra et al., 2020). As a result, the sleep quality of the working population could be further impaired (Garg et al., 2020; Gualano et al., 2020; Yang et al., 2020).

Sleep quality is defined as one’s satisfaction with sleep experience, sufficient sleep quantity, uninterrupted sleep and the feeling of rest and restoration upon awakening (Kline, 2013). The subjective sleep quality can be measured by self-reported methods that use tools such as the Pittsburgh Sleep Quality Index (PSQI; Buysse et al., 1989). The relevance and crucial role of good sleep quality among working adults becomes clearer in both the short and long terms (Chattu et al., 2018). As short-term consequences, the loss of sleep quality could lead to a loss in productivity, road or work-related accidents, absenteeism, negative mood, impaired cognitive function and income loss (Rosekind et al., 2010). In contrast, the long-term effects of it are associated with increased risk and development of chronic noncommunicable diseases such as cardiovascular, obesity, type 2 diabetes mellitus and mental illness.

During the lockdown period of the COVID-19 pandemic, many studies have been conducted by researchers worldwide to measure the sleep quality of different populations (Tasnim et al., 2020). A study by Žilinskas et al. (2022) found that 43% of white-collar workers had poor sleep quality during the lockdown period, 68.9% of participants from general population had poor sleep quality in the study done in United Kingdom (Kantor et al., 2022), 14.9% of the employees who returned to work in China after the lockdown (Yang et al., 2020) had poor sleep quality during the initial period of pandemic and as high as 78.8% among healthcare workers from Kuwait in a study done by Abbas et al. (2021). Studies exploring sleep quality in Malaysia have focused on university students and healthcare workers (Hazizul Hasan and AE Moustafa, 2022), whereas others have elaborated extensively on the psychological impacts of the pandemic on the working population (Abdul Latif et al., 2022). This gap in the literature has yet to be explored in the context of the working population in Malaysia. The factors affecting sleep may differ between healthcare workers and the general working population as well. While healthcare workers’ concern tends more toward fear of contracting infection and isolation from family (Troisi et al., 2021), ordinary workers may lose sleep over job instability, impact on income, finances and lifestyle (Martínez-de-Quel et al., 2021) because of changes in working arrangements during the MCO period. Exposure to such stressors may contribute to triggering anxiety, negatively impacting sleep quality (Alwhaibi and Al Aloola, 2023). Moreover, the literature also states that the sleep quality of the working population can be influenced by socioeconomics (Anders et al., 2014), occupational (Marzo et al., 2021) and lifestyle factors (Madrid-Valero et al., 2017; Touyz et al., 2020; Jeong et al., 2021; Micheletti Cremasco et al., 2021; Robillard et al., 2021).

In times of crisis, widespread disruption to the sleep quality of working adults can have detrimental effects not only on individuals’ personal and family lives but also on work, productivity and society as a whole. As such, it is important to understand the sleep quality status and characteristics associated with poor sleep quality of working people with affected sleep quality as the well-being of this population is critical in regaining momentum in the economy in times of crisis. Investing in this is essential for developing targeted interventions and will enable relevant stakeholders to work collaboratively to implement strategies that mitigate the impact of the crisis on sleep quality, thus fostering the well-being of the Malaysian workforce. This study aims to explore whether socioeconomic, occupational and lifestyle factors are related to the sleep quality of working adults in Malaysia. Resource allocation for public health interventions for the well-being and disease prevention of the target population can be done based on the findings from this research.

This study aims to explore whether socioeconomic, occupational and lifestyle factors are related to the sleep quality of working adults in Malaysia. Resource allocation for public health interventions for the well-being and disease prevention of the target population can be done based on the findings from this research.

## Materials and methods

### Study setting and study population

Study was conducted in January 2021 among working adults in Malaysia during the COVID-19 pandemic. Working adults consisted of those who were employed in any sectors or self-employed, or conducted their own business. The participants’ ages ranged from 18 to 65 years old were included. Those with existing medical conditions, such as long-standing illness and sleep disorders, and those with mental health disorders, part-time workers and shift workers were excluded from the study.

### Study design and procedure

A cross-sectional study using an online survey purposive sampling was conducted by researchers from a medical university for 2 weeks in January 2021 to recruit eligible participants across Malaysia into the study. The sample size was calculated using the Raosoft online calculator, for the estimated large population size of 20,000 and above, a margin of error 5% and confidence interval of 95%. The final sample size was calculated to be 500 after a 35% nonresponse rate was added. The online survey was carried out by sending the Google survey link through Facebook, Instagram and WhatsApp, which brought the participants to the online questionnaire. Since it was during COVID-19 pandemic, the researchers approached the potential eligible participant via these platforms. Study information, which was also stated in the online questionnaire, was explained before obtaining consent on the online form. The questionnaire was pilot tested prior to actual data collection to test the clarity and appropriateness.

### Study instruments

A structured questionnaire composed of four sessions was used to collect the data. Section (A) includes the question items to measure the sociodemographic variables, section (B) measures the lifestyle factors affected during the COVID-19 pandemic and section (C) measures the changes that took place in occupational factors during the COVID-19 pandemic. Section D consists of the tool to measure sleep quality, which is the PSQI. Independent variables were measured in sections A, B and C, whereas section D measured the outcome, sleep quality. PSQI is a self-rated questionnaire examining sleep quality and disturbances of the participants over a month period. Nineteen items generate seven-part scores: sleep latency, duration and disturbances, subjective sleep quality, habitual sleep efficiency, daytime dysfunction and use of sleep medication (Buysse et al., 1989). The tool has a sensitivity of 89.6% and a specificity of 86.5% in differentiating bad and good sleepers. A global PSQI score ranges from 0 to 21. The total PSQI of 5 or more is suggestive of poor sleep (Buysse et al., 1989)

### Data analysis plan

The data collected are tabulated and analyzed by using the Statistical Package for Social Sciences version 26.0. Characteristics of the participants are described by using descriptive statistics such as mean, standard deviation and minimum/maximum for numerical variables and frequency and proportion for categorical variables. The association between the independent variables and the dependent variable is tested by using the chi-square test. Multiple logistic regression analysis is subsequently performed to study the independent effect of variables over the outcome. A p value less than 0.05 will be considered statistically significant.

## Results

### Characteristics of the respondents

Most of the participants in this study were females 331; 66.2 %). The mean age of the participants is 37.5 years (SD = 12.2, min–max = 18–64). In terms of ethnicity, the majority of the respondents who responded to the survey were Chinese (68.6%), followed by Indian (16.6%), Malay (11.4%) and others (3.4%). About half of them were married (51%) and (35.2%) were single, (12.6%) responded as they were in a relationship and (1.0%) reported as divorced (Table 1). The average number of people the respondents supported is 1.7 people (SD = 1.8, min–max = 0–9) (Table 2).

**Table 1.** Characteristics of the respondents

Characteristics	Categorical variables	Subcategories	Frequency (*N* = 500)	Percentage (%)
Outcome variable	Sleep quality	Poor sleep quality	297	59.4
		Normal sleep quality	203	40.6
Sociodemographic factors	Gender	Female	331	66.2
		Male	169	33.8
	Age group (years)	Below 35 years	255	51.0
		35 years and above	245	49.0
	Ethnicity	Malay	57	11.14
		Chinese	343	68.6
		Indian	83	16.6
		Others	17	3.4
	Marital status	Single	176	35.2
		In a relationship	63	12.6
		Married	256	51.2
		Divorced	5	1.0
Socioeconomics factors	Income category	B40	245	49.0
		M40	187	37.4
		T20	68	13.6
	Spending habits changes	Spend more	145	29.0
		Spend less	192	38.4
		No change	163	32.6
	Changes in income	Increased	29	5.8
		Decreased	127	25.4
		No change	344	68.8
Lifestyle factors	Eating habits	Increased intake	102	20.4
		Decreased intake	45	9.0
		Irregular	89	17.8
		No change	264	52.8
	Physical activity changes	More sedentary	106	21.2
		Slightly sedentary	151	30.2
		No change	130	26.0
		Slightly more active	71	14.2
		More active	42	8.4
	Uses of electronic device before sleep	Yes	465	93.0
		No	35	7.0
	Caffeine intake changes	Increased	83	16.6
		No change	235	47.0
		Decreased	60	12.0
		I do not take caffeinated drinks	122	24.4
	Changes in smoking	Increased	19	3.8
		No change	81	16.2
		Decrease	18	3.6
		Not applicable	341	68.2
	Changes in alcohol consumption	Increase	24	4.8
		No change	101	20.2
		Decrease	58	11.6
		Not applicable	277	55.4
Occupational factors	Work arrangement	Work from home	176	35.2
		Office	129	25.8
		Both	195	39.0
	Change in routine	Yes	358	71.6
		No	142	28.4
	Distracted while working	Yes	287	57.4
		No	213	42.6
	Changes in workload	Increased drastically	61	12.2
		Increased	169	33.8
		No change	173	34.6
		Decreased	87	17.4
		Decreased drastically	10	2.0
	Worried about job security	Yes	218	43.6
		No	282	56.4

**Table 2.** Description of numerical variables

Continuous variable	Mean	Median	±Standard deviation	Minimum–maximum
PSQI score	5.6	5.0	2.9	0–16
Age (in years)	37.5	35.0	12.6	18–64
Number of people supporting at home	1.7	2.0	1.8	0–9

With regards to socioeconomic characteristics, almost half of them (49.0%) are from the B40 category. 38% of the respondents stated they spent less during MCO; and the majority (68.8%) responded as no change in income. Looking at the lifestyle factors, there was a small proportion of respondents (9%) who had reduced their eating habits but half of them (52.8%) stated no change. 21.2% and 30.2% of participants expressed getting more sedentary and slightly sedentary respectively during MCO. The majority of them (93%) agreed that they used the electronic devices before sleep. For the caffeine intake, a minority (16.6%) reported increased intake, while a small proportion of respondents, 3.8% and 4.8%, said increase in smoking and alcohol consumption during MCO. With regard to occupational factors, during the time of MCO, 35.2% solely worked from home, while 39% had to work both from home and the office. A clear majority of them (71.6%) said their work routine had changed. More than half (57.4%) said they were distracted with household matters while working during the MCO period. Then, 12.2% and 33.8% reported an increase in workload drastically and increased workload, respectively, and 43.6% reported they were worried about their job security at the same time.

**Figure 1.**
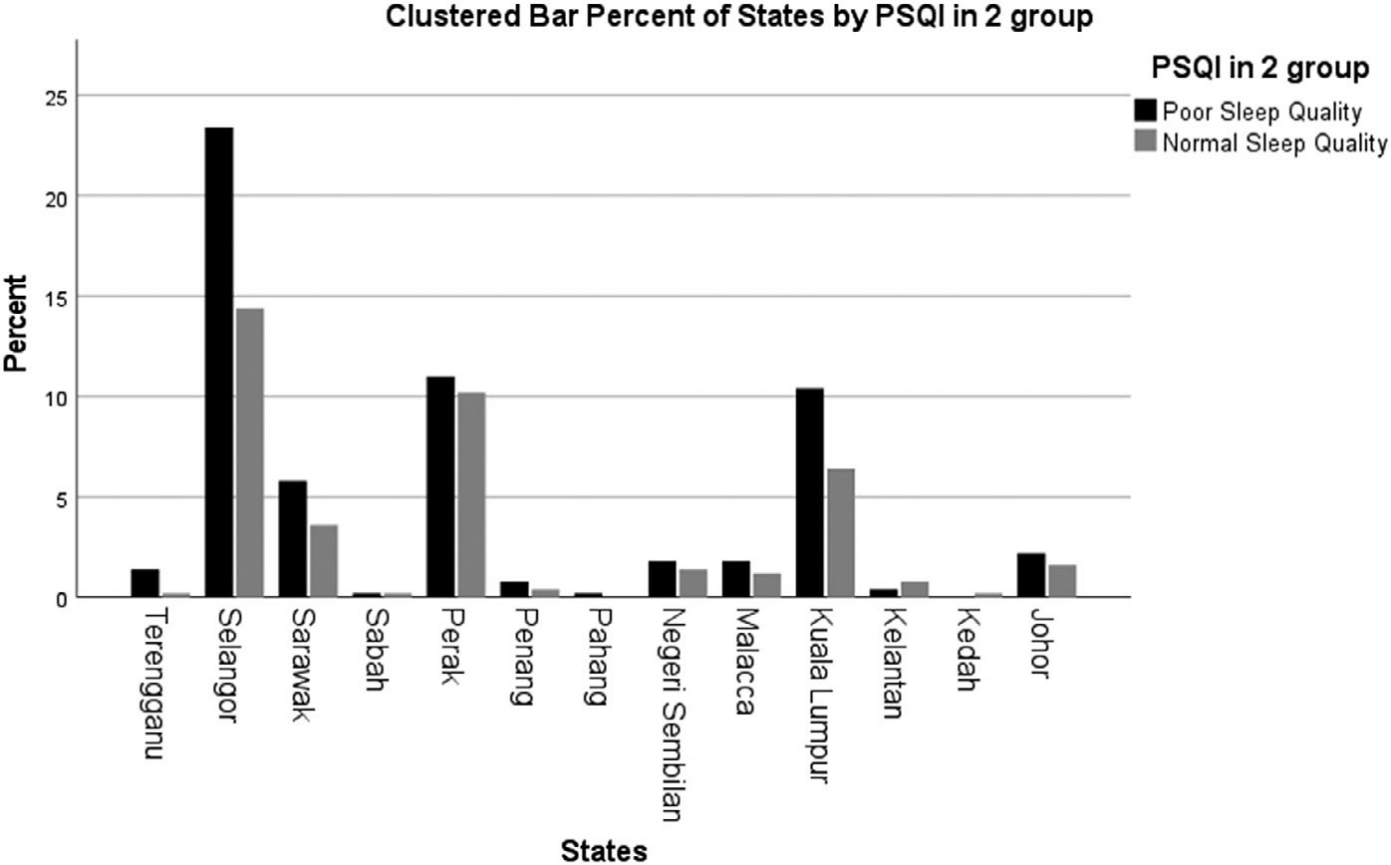
Geographical distribution of respondents with poor sleep quality.

Figure 1 shows that the participants responded to the questionnaire were from 13 different states of Malaysia. Higher numbers of respondents from Kuala Lumpur, Selangor, Malacca, Penang, Johor, Negeri Sembilan, Sarawak, Perak and Terengganu are found to have poorer sleep quality compared to those from other states.

### Sleep quality of the study population

The results show that most of the respondents (59.4%) have poor sleep quality (Table 1). The mean PSQI score of the participants is calculated to be 5.6 (SD = **±**2.9, min–max = 0–16) (Table 2).

Chi-square analysis is performed to test the association of socioeconomic, lifestyle and occupational factors with poor sleep quality in comparison to those who had good sleep quality. The results presented in Table 3 show that changes in spending habit during MCO (*p*-value = 0.003), changes in eating habits (*p*-value = 0.003), changes in physical activity (*p*-value = 0.001), use of electronic devices before sleep (*p*-value = 0.002), increased in smoking (*p*-value = 0.014), changes in workload (*p*-value = 0.001), distractions while working (*p*-value = 0.001) and worries about job security (*p*-value = 0.013) during COVID-19 pandemic are found to be significantly associated with poor sleep quality. The percentages of changes that took place in socioeconomic, lifestyle and occupational categories are also higher in those who had poor sleep quality.

**Table 3.** Socioeconomic, lifestyle and occupational factors associated with sleep quality among the working adult population

Independent variables	Subcategories	Poor sleep quality (*N*_1_ = 297) *n*_1_ (%)	Normal sleep quality (*N*_2_ = 203) *n*_2_ (%)	Total surveyed (*N* = 500) *n* (%)	OR (unadjusted)	95% CI	*p*-Value
Income category	B40	143 (58.4)	102 (41.6)	245 (100)	1.04	0.6–1.79	0.88
	M40	115 (61.5)	72 (38.5)	187 (100)	1.19	0.68–1.09	0.55
	T20	39 (57.4)	29 (42.6)	68 (100)	1		0.75
Spending habit	Spend more	103 (71.0)	42 (29.0)	145 (100)	2.19	1.37–3.52	0.001*
	Spend less	108 (56.3)	84 (43.8)	192 (100)	1.15	0.76–1.75	0.51
	No change	86 (52.8)	77 (47.2)	163 (100)	1		0.003*
Changes in income	Increased	15 (51.7)	14 (48.3)	29 (100)	0.701	0.33–1.49	0.36
	Decreased	74 (58.3)	53 (41.7)	127 (100)	0.91	0.6–1.38	0.67
	No change	208 (60.5)	136 (39.5)	344 (100)	1		0.627
Eating habit	Decreased intake	28(62.2)	17 (37.8)	45 (100)	1.53	0.79–2.92	0.201
	Increased intake	73 (71.6)	29 (28.4)	102 (100)	2.33	1.42–3.82	0.001*
	Irregular	59 (66.3)	30 (33.7)	89 (100)	1.82	1.10–3.01	0.019*
	No change	137 (46.1)	127 (62.6)	264 (100)	1		0.003*
Physical activity	More active	65 (57.5)	48 (42.5)	113 (100)	1.73	1.04–2.89	0.034*
	More sedentary	175 (68.1)	82 (31.9)	257 (100)	2.73	1.78–4.22	0.001*
	No change	57 (43.8)	73 (56.2)	130 (100)	1		0.001*
Use of electronic devices before sleep	Yes	285 (61.3)	180 (38.7)	465 (100)	3.04	1.47–6.25	0.003*
	No	12 (34.3)	23 (65.7)	35 (100)	1		0.002*
Changes in caffeine intake	Increased	60 (72.3)	23 (27.7)	83 (100)	1.81	0.99–3.3	0.053
	No changed	130 (55.3)	105 (44.7)	235 (100)	0.86	0.55–1.34	0.504
	Decreased	35 (58.3)	25 (41.7)	60(100)	0.98	0.52–1.82	0.93
	Not applicable	72 (59.0)	50 (41.0)	122 (100)	1		0.06
Changes in smoking	Increased	17 (89.5)	2 (10.5)	19 (100)	6.44	1.47–28.3	0.014^†^
	No change	49 (60.5)	32 (39.5)	81 (100)	1.16	0.7–1.9	0.56
	Decreased	9 (50)	9 (50)	18 (100)	0.76	0.29–1.96	0.57
	Not applicable	194 (56.9)	147 (43.1)	341 (100)	1		0.08
Changes in alcohol consumption	Increased	15 (62.5)	9 (37.5)	24 (100)	1.22	0.52–2.89	0.65
	No change	60 (59.4)	41 (40.6)	101 (100)	1.07	0.67–1.7	0.77
	Decreased	35 (60.3)	23 (39.7)	58 (100)	1.11	0.63–1.98	0.72
	Not applicable	160 (57.8)	117 (42.2)	277 (100)	1		0.96
Working arrangement	Work from home	108 (61.4)	68 (38.6)	176 (100)	1.29	0.82–2.06	0.27
	Home and office	118 (60.5)	77 (39.5)	195 (100)	1.25	0.79–1.96	0.33
	Office	71 (55.0)	58 (45.0)	129 (100)	1		0.49
Change in routine	Yes	230 (64.2)	128 (35.8)	358 (100)	2.01	1.36–2.98	0.001*
	No	67 (47.2)	75 (52.8)	142 (100)	1		0.001*
Change in workload	Increased	162 (70.4)	68 (29.6)	230 (100)	2.3	1.53–3.47	0.001*
	Decreased	47 (48.5)	50 (51.5)	97 (100)	0.91	0.55–1.49	0.7
	No change	88 (50.9)	85 (49.1)	173 (100)	1		0.001*
Distracted while working	Yes	200 (69.7)	87 (30.3)	287 (100)	2.75	1.9–3.98	0.001*
	No	97 (45.5)	116 (54.5)	213 (100)	1		0.001*
Worries about job security	Yes	143 (65.6)	75 (34.4)	218 (100)	1.59	1.1–2.28	0.013*
	No	154 (54.6)	128 (45.4)	282 (100)	1		0.013*

* Here, *p*-value of <0.05 was considered as significant.

† Fisher’s exact test, B40 = below 40%, M40 = middle 40%, T20 = top 20% of the income status in Malaysia.

Table 4 summarizes the predictor factors associated with poor sleep quality among the participants. A total of 10 variables that had a p-value of less than 0.25 were selected from the chi-square analysis to conduct the multiple logistic regression analysis. After logistic regression analysis was performed, it was identified that the participants who used electronic devices before sleep (OR = 2.33, 95% CI = 1.02–5.35, *p*-value = 0.046), those who had increased amount of workload (OR = 0.45, 95% CI = 0.26–0.79, *p*-value = 0.005), those who had decreased amount of workload (OR = 0.48, 95% CI = 0.29–0.78, *p*-value = 0.003), and the participants who were distracted with family matters while working (OR = 0.57, 95% CI = 0.37–0.89, *p*-value = 0.014) were found to predict poor sleep quality during the COVID-19 pandemic in our study population.

**Table 4.** Multiple logistic regression analysis of poor sleep quality among the working adult population

Independent variables	Subcategories	B coefficient	Standard error	OR (adjusted)	95% CI	*p*-Value
Spending habit	Spend more	–0.507	0.273	0.60	0.35–1.03	0.063
	Spend less	–0.311	0.281	0.73	0.42–1.27	0.267
	No change			1		
Eating habit	Decreased intake	0.394	0.436	1.48	0.63–3.49	0.366
	Increased intake	–0.174	0.428	0.84	0.36–1.95	0.685
	Irregular	–0.138	0.381	0.87	0.41–1.84	0.718
	No change			1		
Physical activity	More active	0.171	0.264	1.19	0.71–1.99	0.518
	More sedentary	–0.402	0.307	0.67	0.37–1.22	0.190
	No change			1		
Use of electronic devices before sleep	Yes	–0.846	0.423	0.43	0.19–0.98	0.046*
	No			1		
Changes in caffeine intake	Increased	–0.247	0.331	0.78	0.41–1.49	0.455
	No changed	–0.288	0.414	0.75	0.33–1.69	0.487
	Decreased	–0.396	0.353	0.67	0.34–1.34	0.262
	Not applicable			1		
Changes in smoking	Increased	–0.922	0.818	0.39	0.08–1.97	0.260
	No change	–1.329	0.949	0.27	0.04–1.70	0.161
	Decreased	–1.089	0.790	0.34	0.07–1.58	0.168
	Not applicable			1		
Change in routine	Yes	–0.160	0.252	0.85	0.52–1.39	0.526
	No			1		
Change in workload	Increased	–0.792	0.284	0.45	0.26–0.79	0.005*
	Decreased	–0.729	0.246	0.48	0.29–0.78	0.003*
	No change			1		
Distracted while working	Yes	–0.605	0.225	0.57	0.37–0.89	0.014*
	No			1		
Worries about job security	Yes	–0.316	0.221	0.73	0.47–1.12	0.154
	No			1		

* Here, *p*-value of <0.05 was considered as significant.

## Discussion

### Prevalence

The prevalence of poor sleep quality among the working adults during the MCO period of COVID-19 pandemic in Malaysia is 59.4%, which is found to be high. The high prevalence was also seen in the studies done by Abbas et al. (2021), Kantor et al. (2022) and Žilinskas et al. (2022), which are 78.8%, 68.9% and 43%, respectively. However, in a study from China by Yang et al. (2020), the prevalence was lower, 14.9%. The higher proportion found in this study may be explained by the condition where the people are bound to stay indoors during a crisis like COVID-19 pandemic. Sociodemographic factors also explain the findings since majority of the participants were females and were married which was found to be normally associated with poorer sleep quality (Madrid-Valero et al., 2017)

Another reason could be the stressful situation, which was not measured in this study, that working adults went through due to the sudden and unexpected changes in socioeconomic, lifestyle and working arrangements that took place in the majority of the participants of this study. The high prevalence of poor sleep quality could also be due to selection bias as most of the participants recruited into the study were from Selangor and Kuala Lumpur, the two states where the working populations are highest and also the states that were worse hit by COVID-19 (Hashim et al., 2021) during the pandemic.

### Factors associated with poor sleep quality among working adults during COVID-19 pandemic

#### Socioeconomic factors

The results from this study say that during the MCO period, the employees whose spending habit has increased is significantly associated with poor sleep quality in the initial chi-square analysis. However, it is no longer significant in the regression analysis. Even though, the results are not significant for the socioeconomic factors in this study, socioeconomic factors indeed have proven to impact sleep quality. Changes in spending habits such as spending more may impose a financial strain on the individual (Abdul Latif et al., 2022). Government financial support packages and wage subsidy programs implemented as part of the aid and relief efforts for the COVID-19 pandemic (*Wage Subsidy* Program, 2021) might have absorbed the initial financial strain among the employees which explained the insignificant results under socioeconomic factors.

#### Lifestyle factors

The findings also indicate that lifestyle factors have effects on the PSQI score. In the initial chi-square analysis, employees who increased in eating habit, got more sedentary, used electronic devices before sleep, increased in caffeine intake and smoking were found to be significantly associated with poor sleep quality. However, after the multiple logistic regression analyses, only one variable, the use of electronic devices before sleep, shows significant associations with poor sleep quality. It is also a well-studied fact that using electronic devices before causes a delay in melatonin secretion and disrupts sleep (Randjelović et al., 2019; Boukhris et al., 2023). The increased use of electronic devices in the time of MCO must have contributed to this process putting people at risk of poor sleep quality.

#### Occupational factors

Besides that, occupational factors such as changes in workload and getting distracted while working had an impact on one’s sleep quality. According to research (Conroy et al., 2021), since the working adults were restrained at home during the pandemic, this inevitably disrupted their usual daily routine being disrupted, making them more prone to the various distractions at home from various sources thereby affecting their work. This might result in putting one at risk of getting more stressed and feeling overwhelmed by the escalating amount of workload that is left undone which in turn causing the poor sleep quality.

## Conclusions

Our findings suggest that the proportion of poor sleep quality among working adults is high during the MCO period in Malaysia. The socioeconomic factors are not associated with poor sleep quality among the participants. The variables such as the use of electronic devices before sleep, changes in the amount of workload and being distracted while working during the MCO period of the COVID-19 pandemic have caused them to be significantly associated with poor sleep quality, predicting this outcome among the working population in Malaysia.

### Recommendation

The results highlight that during the period of crisis like COVID-19 pandemic, there is a need for public health interventions that aim to empower the working population to adopt a healthy lifestyle, particularly sleep hygiene education on the use of electronic devices before sleep to improve sleep quality. The findings also provide employers with the evidence to implement additional measures to support employees’ well-being and to provide a healthy workplace during the challenging times. Policy recommendations are also made to implement flexible working arrangements, workload management, workplace mental health support and legal protections for reasonable working hours, rest breaks and time off during crises. Post-pandemic studies on sleep quality and its impact on the physical and mental health of the working population are recommended to explore the health needs of the working population.

### Limitations

Probability sampling methods could not be deployed in this study, which might impact its generalizability even though the study covered all states in Malaysia. The independent variables are measured subjectively, such as changes in lifestyle factors and occupational factors; hence, the accuracy of the level of changes might be affected. The study included more females who are more vulnerable to poor sleep quality than men, and potential selection bias during the recruitment process, contributing to high proportion of poor sleep quality in this study. The accuracy of the results can also be affected by recall bias, which is a systematic error that occurs when participants do not remember the previous experience accurately.

## Supporting information

Aye and Lee supplementary material Aye and Lee supplementary material

## Data Availability

Data collected for this project, in the form of raw data in Excel, are available upon request.

## Supplementary material

The supplementary material for this article can be found at http://doi.org/10.1017/gmh.2024.23.
