# Supplementary material for: Poor sleep quality and its associated factors among working adults during COVID-19 pandemic in Malaysia
Source: Glob Ment Health (Camb). 2024 Mar 13;11:e28. doi: 10.1017/gmh.2024.23 (PMC10988144; doi:10.1017/gmh.2024.23)
Supplement: Aye and Lee supplementary material [file S2054425124000232sup001.docx]

Reviewer(s)' Comments to Author:

Reviewer: 1

Comments to the Author
No further comments. Good job from the authors.

Reviewer: 2

Comments to the Author

| **Title:** To omit Cross-sectional | - This was from the first round of comments. The author overlooked and did not respond to it. - The term “cross-sectional study” is omitted. |
| --- | --- |
| **Methodology:** p6 line115 The statement seems not complete… “…was conducted by researchers from a Medical for…” . Which part of Medical? | - P6 line 115: Inserted a Medical University. |
| p6 line116 “…to recruit 500 eligible participants..”. the “500” should be removed. | - P6 line116: “500” is removed. |
| p6 line118 “…estimated population size of 20,000..” please elaborate on where and how the estimated working population size was 20,000? | - P6 lines 117-118: this was rewritten as “for the large population size of 20,000 and above”. - The reason is the sample size doesn’t change much after 20,000 |
| What is the nature of the sampling procedure? | - P6 line 115: The term “purposive sampling” is inserted. - Line 120: The term “online survey” is mentioned. |
| As for the current study, how would the sleep quality be categorized? Need to describe in details on the procedure of logistic method. | - Line 144: PSQI score above 5 is considered poor sleep quality by Byusse et.al, 1989. - Line 122-124: Added “Since it was during the COVID-19 pandemic, the researchers approached the potential eligible participant via these platforms. Study information, which was also stated in the online questionnaire, was explained before obtaining consent on the online form.” |
| **Results:**  Should report how many responds received? Out of this, how many of the responses were complete and proceed for further analysis? This will give how the percentages of respondents. | - Actual number of participants approached is not known since there were scenarios where the researchers posted the link in online groups and social media. Hence, the response rate is not known. |
| p7 line152  The result for the “Sleep Quality of the Study Population” should place after the “Characteristics of the Respondents” | - It is moved to line 187-190 after “characteristics” |
| p10 line 208 The term “predict” is incorrectly use as the analysis was looking at an “odds ratio”, hence, the appropriate term to be used is “associate” | - Line 208: The term “predict” is replaced with “factors associated with”. |
| p10 line 286 - 289 “The variables such as the use of electronic devices before sleep, changes in the amount of workload, and being distracted while working during the MCO period of the COVID-19 pandemic have caused them to be significantly associated with poor sleep quality, predicting this outcome among the working population in Malaysia” | - Line 294-297: The term “predicting this outcome” is removed. |
| “Figure 1: Geographical distribution of respondents with poor sleep quality” More appropriate title: **Figure 1: Respondents with sleep quality patterns (poor and good) according to the states in Malaysia.** | - Changed into suggested title. |
| “Table 3: Socio-Economic, Lifestyle, and Occupational Factors Associated with Poor Sleep Quality” More appropriate title: **Table 3: Socio-Economic, Lifestyle, and Occupational Factors Associated with Sleep Quality Among the Working Adult Population.** | - Changed into suggested title. |
| The term “Normal sleep quality” in Table 3 is not consistent with “Good Sleep Quality” in Figure 1. | - The term is corrected in Figure 1 to be aligned with the term in table 3. |
| “Table 4: Multivariate Logistic Regression Analysis of Predictors of Poor Sleep Quality” More appropriate title: **Table 4: Multiple Logistic Regression Analysis of Poor Sleep Quality Among the Working Adult Population** | - Changed into suggested title. |
| Limitation:  The limitation did not discuss about the recalled biases. | - Line 328: The limitation on recalled bias is added. |
