# Supplementary material for: Poor sleep quality and its associated factors among working adults during COVID-19 pandemic in Malaysia
Source: Glob Ment Health (Camb). 2024 Mar 13;11:e28. doi: 10.1017/gmh.2024.23 (PMC10988144; doi:10.1017/gmh.2024.23)
Supplement: Peer review history

# Editor report: Recommendation: Poor sleep quality and its associated factors among working adults during COVID-19 pandemic in Malaysia — R0/PR2

**DOI:** 10.1017/gmh.2024.23.pr2

**Affiliations:** School of Public Health and Preventive Medicine, Monash University Faculty of Medicine Nursing and Health Sciences, Australia

The reviewers commend your paper but each have recommendations to strengthen it. Please address all the reviewers' comments.

# Editor report: Decision: Poor sleep quality and its associated factors among working adults during COVID-19 pandemic in Malaysia — R0/PR3

**DOI:** 10.1017/gmh.2024.23.pr3

**Affiliations:** London School of Hygiene & Tropical Medicine, United Kingdom of Great Britain and Northern Ireland

No accompanying comment.

# Editor report: Recommendation: Poor sleep quality and its associated factors among working adults during COVID-19 pandemic in Malaysia — R1/PR5

**DOI:** 10.1017/gmh.2024.23.pr5

**Affiliations:** School of Public Health and Preventive Medicine, Monash University Faculty of Medicine Nursing and Health Sciences, Australia

No accompanying comment.

# Editor report: Decision: Poor sleep quality and its associated factors among working adults during COVID-19 pandemic in Malaysia — R1/PR6

**DOI:** 10.1017/gmh.2024.23.pr6

**Affiliations:** London School of Hygiene & Tropical Medicine, United Kingdom of Great Britain and Northern Ireland

No accompanying comment.

# Editor report: Recommendation: Poor sleep quality and its associated factors among working adults during COVID-19 pandemic in Malaysia — R2/PR8

**DOI:** 10.1017/gmh.2024.23.pr8

**Affiliations:** School of Public Health and Preventive Medicine, Monash University Faculty of Medicine Nursing and Health Sciences, Australia

No accompanying comment.

# Editor report: Decision: Poor sleep quality and its associated factors among working adults during COVID-19 pandemic in Malaysia — R2/PR9

**DOI:** 10.1017/gmh.2024.23.pr9

**Affiliations:** London School of Hygiene & Tropical Medicine, United Kingdom of Great Britain and Northern Ireland

No accompanying comment.
